# ASO Author Reflections: 90-Day Postoperative Complications After Total or Partial Gastrectomy with Antecolic Versus Retrocolic Reconstruction

**DOI:** 10.1245/s10434-024-15560-x

**Published:** 2024-06-07

**Authors:** Anna Junttila, Olli Helminen, Joonas H. Kauppila, the FINEGO Group

**Affiliations:** 1 Division of Digestive Surgery and Urology, Turku University Hospital, Turku, Finland; 2 Surgery Research Unit, Medical Research Center Oulu, Oulu University Hospital and University of Oulu, Oulu, Finland; 3 Upper Gastrointestinal Surgery, Department of Molecular Medicine and Surgery, Karolinska Institutet and Karolinska University Hospital, Stockholm, Sweden

## Past

The studies comparing antecolic and retrocolic reconstruction have mainly focused on patients undergoing bariatric surgery or pancreaticoduodenectomy. The reconstruction after open total or partial gastrectomy often has been performed via retrocolic route, because it results with less tension due to shorter route compared with antecolic reconstruction. After implementation of laparoscopic surgery, the antecolic route has gained popularity for its technical simplicity, and it is more commonly used in open approach as well.^1^ The purpose of the present study was to examine 90-day anastomotic and other postoperative complications in gastric cancer patients after total or partial gastrectomy with antecolic versus retrocolic reconstruction in a population-based setting.

## Present

We conducted a population-based, nationwide cohort study including all 2,063 gastric adenocarcinoma patients undergoing total or partial gastrectomy (antecolic reconstruction n = 814 and retrocolic reconstruction n = 1249) in Finland in 2005–2016.^2^ Logistic regression adjusted for confounders provided odds ratios (OR) with 95% confidence intervals (CI) of 90-day mortality. Anastomotic complication rate was 3.8% with antecolic and 5.0% with retrocolic reconstruction, but this difference was not statistically significant after adjustment. In subgroup analysis of total gastrectomy patients, the risk of major complications was lower with antecolic compared with retrocolic reconstruction.

## Future

The present study shows that the antecolic reconstruction is not associated to higher risk of anastomotic complications. However, a decreased risk of major complications is seen after total gastrectomy compared to retrocolic reconstruction. Because of these findings and technical simplicity of antecolic reconstruction, it should be the preferred reconstruction route.

## References

[1] Ikeda M, Yoshida M, Mitsumori N, Etoh T, Shibata C, Terashima M (2022). Assessing optimal Roux-en-Y reconstruction technique after total gastrectomy using the Postgastrectomy Syndrome Assessment Scale-45. World J Clin Oncol..

[2] Junttila A, Helminen O, Helmiö M (2024). Comparison of postoperative complications after gastrectomy for gastric cancer with antecolic versus retrocolic reconstruction: a population-based study. Ann Surg Oncol..

